# *rac*-3,4-*trans*-Dichloro-1,2,3,4-tetra­hydro-2-naphthyl acetate

**DOI:** 10.1107/S1600536808035563

**Published:** 2008-11-08

**Authors:** Ertan Şahin, Nurhan Kishali, Yunus Kara

**Affiliations:** a Department of Chemistry, Faculty of Science, Atatürk University, 25240-Erzurum, Turkey

## Abstract

The title compound, C_12_H_12_Cl_2_O_2_, has a bicyclic skeleton containing cyclo­hexene and benzene fragments. The cyclo­hexene ring adopts a half-chair conformation with displacements of two atoms out of the least-squares plane of 0.311 (2) and −0.336 (2) Å. The Cl atoms are *trans*-positioned.

## Related literature

For related literature, see: Frimer (1985*a* ▶,*b* ▶); March & Smith (2001 ▶); McBride *et al.* (1999 ▶); Metha & Ramesh (2003 ▶, 2005 ▶); Metha *et al.* (2003 ▶); Patai (1983 ▶); Ros *et al.* (2006 ▶); Wasserman & Murray (1979 ▶). For related structures, see: Kishali *et al.* (2006*a* ▶,*b* ▶).
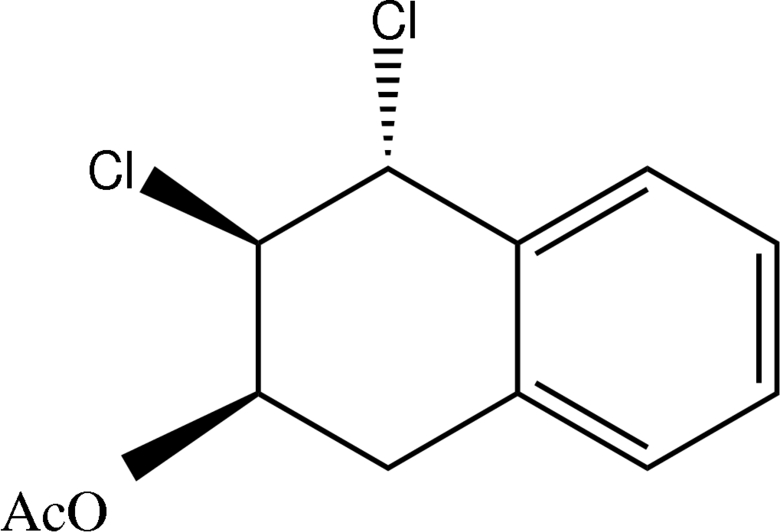


## Experimental

### 

#### Crystal data

C_12_H_12_Cl_2_O_2_
*M* *_r_* = 259.12
Monoclinic, 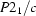

*a* = 12.931 (5) Å
*b* = 12.478 (5) Å
*c* = 7.441 (4) Å
β = 101.040 (5)°
*V* = 1178.4 (9) Å^3^
*Z* = 4
Mo *K*α radiation
μ = 0.53 mm^−1^
*T* = 293 (2) K
0.2 × 0.2 × 0.2 mm

#### Data collection

Rigaku R-AXIS conversion diffractometer
Absorption correction: multi-scan (Blessing, 1995 ▶) *T*_min_ = 0.897, *T*_max_ = 0.898
34473 measured reflections
3627 independent reflections
2486 reflections with *I* > 2σ(*I*)
*R*_int_ = 0.083
25 standard reflections every 200 reflections intensity decay: 3%

#### Refinement

*R*[*F* ^2^ > 2σ(*F* ^2^)] = 0.063
*wR*(*F* ^2^) = 0.154
*S* = 1.09
3627 reflections
146 parameters
H-atom parameters constrained
Δρ_max_ = 0.21 e Å^−3^
Δρ_min_ = −0.34 e Å^−3^

### 

Data collection: *CrystalClear* (Rigaku/MSC, 2005 ▶); cell refinement: *CrystalClear*; data reduction: *CrystalClear*; program(s) used to solve structure: *SHELXS97* (Sheldrick, 2008 ▶); program(s) used to refine structure: *SHELXL97* (Sheldrick, 2008 ▶); molecular graphics: *ORTEP-3 for Windows* (Farrugia, 1997 ▶); software used to prepare material for publication: *WinGX* (Farrugia, 1999 ▶).

## Supplementary Material

Crystal structure: contains datablocks global, I. DOI: 10.1107/S1600536808035563/kp2181sup1.cif

Structure factors: contains datablocks I. DOI: 10.1107/S1600536808035563/kp2181Isup2.hkl

Additional supplementary materials:  crystallographic information; 3D view; checkCIF report

## Figures and Tables

**Fig. 1.**
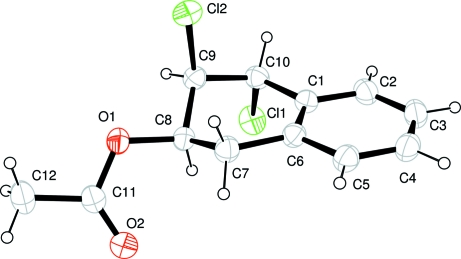
Molecular structure of I with the atom-numbering scheme. The displacement ellipsoids are drawn at the 50% probability level.

**Fig. 2.**
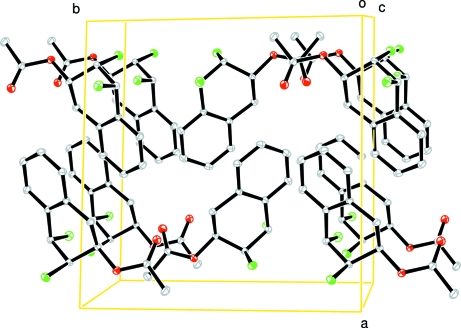
A packing diagram. H atoms have been omitted for clarity.

**Fig. 3.**
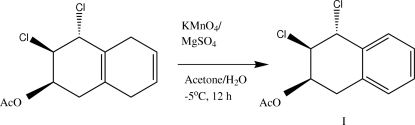
The preparation of the title compound.

## supplementary crystallographic information

### Comment

Oxyfunctionalization of organic unsaturated molecules are efficiently performed by an oxygen atom. It has been reported that the main reactions of singlet oxygen are cycloaddition and ene-reaction (Frimer, 1985*a*,*b*; Patai 1983; Wasserman & Murray, 1979). We have successively used isotetralin (1,4,5,8-tetrahydronaphthalene) for a short and stereocontrolled synthesis of a new class of bis-endoperoxide (Kishali *et al.*, 2006*a*) and the interesting chlorination product, (2*S**,3*S**,4*S**)-3,4-dichloro-1,2,3,4,5,8-hexahydro naphthalen-2-yl acetate (Kishali *et al.*, 2006b), *cf.* Fig. 3). A multistep procedure for the preparation of a new family of annulated inositols from tetrahydronaphthalene has been developed (Metha & Ramesh, 2003, 2005; Metha *et al.*, 2003). Vicinal halohydrins are versatile building blocks and key intermediates for the synthesis of many bioactive molecules (Ros *et al.*, 2006).

We aimed to synthesize the polyhydroxyhalohidrins from (2*S**, 3S*, 4S*)-3, 4-dichloro-1, 2, 3, 4, 5, 8-hexahydronaphthalen-2-yl acetate with KMnO_4_, but reaction of (2*S**, 3S*, 4S*)-3, 4-dichloro-1, 2, 3, 4, 5, 8-hexahydronaphthalen-2-yl acetate with KMnO_4_ gave a very interesting and unexpected product (I) including an aromatic ring.

Potassium permanganate, a very oxidizing agent can be used to oxidize alkenes to diols (March & Smith, 2001). Limiting the reaction to hydroxylation alone is often difficult, and it is usually attempted by using a cold, diluted and basic KMnO_4_ solution. Potassium permanganate, when supported on alumina and used in acetone, reacts very differently than potassium permanganate in aqueous solution. The synthesis of benzene derivatives from 1,4-cyclohexadienes by using KMnO_4_—Al_2_O_3_ is already known in the literature (McBride *et al.*, 1999). (2*S**, 3S*, 4S*)-3, 4-dichloro-1, 2, 3, 4, 5, 8-hexahydronaphthalen-2-yl acetate was synthesized as described in the literature (Kishali *et al.*, 2006*b*). We expected the formation of a diol from reaction of (2*S**, 3S*, 4S*)-3, 4-dichloro-1, 2, 3, 4, 5, 8-hexahydronaphthalen-2-yl acetate with KMnO_4_, but instead the formation of (I) was detected.

The bicyclic skeleton contains a cyclohexene and a benzene ring sharing a common C?C bond [C1—C6=1.400 (3) Å] (Fig. 1). The Cl atoms are *trans*-positioned. C10—Cl1 and C9—Cl2 bond lengths are 1.827 (3) and 1.802 (3) Å, respectively. The three stereogenic centres C2, C3, and C4 are of the same configuration; during crystallization the racemization occurred. All these values are comparable with our previous structure (C_12_H_14_O_2_Cl_2_) (Kishali *et al.*, 2006*b*), in which, only the difference, hexadien moiety exists instead of benzene in the carbocyclic ring. Crystal packing is dominated by van der Waals contacts.

### Experimental

3,4-Dichloro-1, 2, 3, 4-tetrahydro-naphtahalen-2-yl acetate was prepared as follows. To a magnetically stirred acetone solution (25 ml of (2*S**, 3S*, 4S*)-3, 4-dichloro-1, 2, 3, 4, 5, 8-hexahydronaphthalen-2-yl acetate (260 mg, 1 mmol) was added a solution of KMnO_4_ (158 mg, 1 mmol) and MgSO_4_ (144 mg, 1.2 mmol) in water (20 ml) at 253 K during 5 h. After the addition was completed, the reaction mixture was stirred for an additional 15 h at the given temperature and then filtered. The filtrate was concentrated to 20 ml by evaporation. The aqueous solution was extracted with ethyl acetate (3x30 ml) and the extract were dried (Na_2_SO_4_). Evaporation of the solvent gave racemic mixture of compound I. It was separated by column chromatography, eluting with ethylacetate/hexanes (120 mg, 42%, colourless solid). Colourless solid from CH_2_Cl_2_/hexane. m. p: 348–349 K. ^1^H-NMR (400 MHz, CDCl_3_, p.p.m.): 7.39–7.12 (m, 4H), 5.78 (m, 1*H*-C_(2)_), 5.35 (d, J = 3.3, 1*H*-C_(4)_), 4.74 (t, J = 2.9, 1*H*-C_(3)_), 3.2 (m, 2*H*-C_(1)_), 2.14 (s, 3*H*-C_(Ac)_). ^13^C-NMR (100 MHz, CDCl_3_, p.p.m.):170.4, 132.9, 131.7, 130.9, 129.5, 129.3, 127.6, 67.3, 61.3, 60.1, 30.2, 30.0, 21.3. calcd C 55.62, H 4.67; found C 55.87, H 4.89.

### Refinement

H atoms were placed in geometrically idealized positions (C—H=0.93–0.98 Å) and treated as riding, with *U*_iso_(H)=1.2*U*_eq_(C)(for methine and methylene) or 1.5*U*_eq_(methyl C).

### Crystal data

**Table:**

C_12_H_12_Cl_2_O_2_	*F*(000) = 536
*M**_r_* = 259.12	*D*_x_ = 1.461 Mg m^−^^3^
Monoclinic, *P*2_1_/*c*	Mo *K*α radiation, λ = 0.71073 Å
Hall symbol: -P 2ybc	Cell parameters from 6382 reflections
*a* = 12.931 (5) Å	θ = 2.3–30.6°
*b* = 12.478 (5) Å	µ = 0.53 mm^−^^1^
*c* = 7.441 (4) Å	*T* = 293 K
β = 101.040 (5)°	Needle, pale white
*V* = 1178.4 (9) Å^3^	0.2 × 0.2 × 0.2 mm
*Z* = 4	

### Data collection

**Table:**

Rigaku R-AXIS conversion diffractometer	*R*_int_ = 0.083
dtprofit.ref scans	θ_max_ = 30.7°, θ_min_ = 2.3°
Absorption correction: multi-scan (Blessing, 1995)	*h* = −18→18
*T*_min_ = 0.897, *T*_max_ = 0.898	*k* = −17→17
34473 measured reflections	*l* = −10→10
3627 independent reflections	25 standard reflections every 200 reflections
2486 reflections with *I* > 2σ(*I*)	intensity decay: 3%

### Refinement

**Table:**

Refinement on *F*^2^	0 restraints
Least-squares matrix: full	H-atom parameters constrained
*R*[*F*^2^ > 2σ(*F*^2^)] = 0.063	*w* = 1/[σ^2^(*F*_o_^2^) + (0.0549*P*)^2^ + 0.2956*P*] where *P* = (*F*_o_^2^ + 2*F*_c_^2^)/3
*wR*(*F*^2^) = 0.154	(Δ/σ)_max_ < 0.001
*S* = 1.09	Δρ_max_ = 0.21 e Å^−^^3^
3627 reflections	Δρ_min_ = −0.34 e Å^−^^3^
146 parameters	

### Special details

**Table:**

Geometry. All e.s.d.'s (except the e.s.d. in the dihedral angle between two l.s. planes) are estimated using the full covariance matrix. The cell e.s.d.'s are taken into account individually in the estimation of e.s.d.'s in distances, angles and torsion angles; correlations between e.s.d.'s in cell parameters are only used when they are defined by crystal symmetry. An approximate (isotropic) treatment of cell e.s.d.'s is used for estimating e.s.d.'s involving l.s. planes.

### Fractional atomic coordinates and isotropic or equivalent isotropic displacement parameters (Å^2^)

**Table:**

	*x*	*y*	*z*	*U*_iso_*/*U*_eq_	
Cl1	0.22319 (6)	0.59964 (5)	0.18471 (9)	0.0666 (2)	
Cl2	0.12465 (5)	0.59224 (5)	0.70388 (10)	0.0667 (2)	
O1	0.13535 (12)	0.35895 (13)	0.5577 (2)	0.0535 (4)	
O2	0.23303 (14)	0.22818 (15)	0.4683 (3)	0.0722 (5)	
C1	0.34102 (17)	0.62147 (18)	0.5296 (3)	0.0486 (5)	
C2	0.40917 (19)	0.7052 (2)	0.5078 (3)	0.0580 (6)	
H2	0.3843	0.7623	0.4315	0.07*	
C3	0.5123 (2)	0.7044 (2)	0.5973 (4)	0.0646 (7)	
H3	0.5573	0.7601	0.5804	0.078*	
C4	0.5489 (2)	0.6200 (2)	0.7129 (4)	0.0636 (7)	
H4	0.6184	0.6197	0.7756	0.076*	
C5	0.48316 (19)	0.5365 (2)	0.7357 (3)	0.0577 (6)	
H5	0.5089	0.4802	0.8135	0.069*	
C6	0.37804 (17)	0.53492 (18)	0.6436 (3)	0.0478 (5)	
C7	0.30921 (18)	0.44074 (19)	0.6704 (3)	0.0533 (5)	
H7A	0.3489	0.3748	0.6696	0.064*	
H7B	0.2883	0.4467	0.7884	0.064*	
C8	0.21251 (17)	0.43671 (18)	0.5214 (3)	0.0483 (5)	
H8	0.2343	0.4166	0.4069	0.058*	
C9	0.15676 (17)	0.54420 (18)	0.4929 (3)	0.0496 (5)	
H9	0.0921	0.5369	0.4005	0.06*	
C10	0.22906 (18)	0.62559 (18)	0.4281 (3)	0.0512 (5)	
H10	0.2012	0.6975	0.4416	0.061*	
C11	0.15545 (19)	0.25602 (19)	0.5198 (3)	0.0546 (6)	
C12	0.0677 (2)	0.1837 (2)	0.5466 (4)	0.0707 (7)	
H12A	0.0407	0.2066	0.6519	0.106*	
H12B	0.0934	0.1116	0.5648	0.106*	
H12C	0.0124	0.1865	0.4402	0.106*	

### Atomic displacement parameters (Å^2^)

**Table:**

	*U*^11^	*U*^22^	*U*^33^	*U*^12^	*U*^13^	*U*^23^
Cl1	0.0761 (5)	0.0694 (4)	0.0518 (4)	−0.0055 (3)	0.0059 (3)	0.0081 (3)
Cl2	0.0632 (4)	0.0683 (4)	0.0735 (5)	0.0053 (3)	0.0256 (3)	−0.0070 (3)
O1	0.0468 (9)	0.0490 (9)	0.0664 (10)	−0.0052 (7)	0.0152 (8)	0.0032 (7)
O2	0.0656 (11)	0.0582 (11)	0.0966 (15)	−0.0020 (9)	0.0247 (10)	−0.0128 (10)
C1	0.0485 (12)	0.0474 (11)	0.0511 (12)	−0.0018 (9)	0.0125 (10)	−0.0053 (9)
C2	0.0639 (15)	0.0509 (13)	0.0615 (14)	−0.0085 (11)	0.0179 (12)	−0.0030 (11)
C3	0.0614 (15)	0.0659 (16)	0.0701 (16)	−0.0204 (12)	0.0216 (13)	−0.0162 (13)
C4	0.0460 (13)	0.0794 (18)	0.0653 (16)	−0.0074 (12)	0.0108 (12)	−0.0163 (13)
C5	0.0487 (13)	0.0634 (15)	0.0601 (14)	0.0002 (11)	0.0082 (11)	−0.0047 (11)
C6	0.0444 (11)	0.0518 (12)	0.0482 (12)	−0.0019 (9)	0.0114 (9)	−0.0028 (9)
C7	0.0474 (12)	0.0509 (12)	0.0605 (14)	0.0011 (10)	0.0076 (11)	0.0062 (11)
C8	0.0430 (11)	0.0455 (11)	0.0579 (13)	−0.0031 (9)	0.0131 (10)	0.0016 (10)
C9	0.0438 (11)	0.0513 (12)	0.0532 (12)	0.0022 (9)	0.0083 (10)	−0.0018 (10)
C10	0.0533 (13)	0.0455 (11)	0.0544 (13)	0.0019 (9)	0.0094 (10)	0.0015 (10)
C11	0.0545 (13)	0.0508 (13)	0.0566 (14)	−0.0052 (10)	0.0055 (11)	0.0025 (10)
C12	0.0676 (16)	0.0600 (16)	0.0823 (19)	−0.0171 (13)	0.0092 (14)	0.0069 (14)

### Geometric parameters (Å, °)

**Table:**

Cl1—C10	1.827 (3)	C7—H7A	0.97
Cl2—C9	1.802 (3)	C7—H7B	0.97
O1—C11	1.351 (3)	C8—H8	0.98
O1—C8	1.454 (3)	C10—H10	0.98
C9—C8	1.518 (3)	C4—C5	1.376 (4)
C9—C10	1.520 (3)	C4—C3	1.385 (4)
C9—H9	0.98	C4—H4	0.93
C6—C1	1.400 (3)	C5—H5	0.93
C6—C5	1.401 (3)	C2—C3	1.372 (4)
C6—C7	1.510 (3)	C2—H2	0.93
C1—C2	1.396 (3)	C3—H3	0.93
C1—C10	1.500 (3)	C12—H12A	0.96
O2—C11	1.192 (3)	C12—H12B	0.96
C11—C12	1.493 (3)	C12—H12C	0.96
C7—C8	1.504 (3)		
			
C11—O1—C8	115.45 (18)	C7—C8—H8	108.2
C8—C9—C10	109.28 (18)	C9—C8—H8	108.2
C8—C9—Cl2	110.77 (17)	C1—C10—C9	114.21 (19)
C10—C9—Cl2	108.22 (16)	C1—C10—Cl1	110.26 (16)
C8—C9—H9	109.5	C9—C10—Cl1	106.55 (16)
C10—C9—H9	109.5	C1—C10—H10	108.6
Cl2—C9—H9	109.5	C9—C10—H10	108.6
C1—C6—C5	118.2 (2)	Cl1—C10—H10	108.6
C1—C6—C7	122.58 (19)	C5—C4—C3	120.4 (2)
C5—C6—C7	119.2 (2)	C5—C4—H4	119.8
C2—C1—C6	119.8 (2)	C3—C4—H4	119.8
C2—C1—C10	119.1 (2)	C4—C5—C6	121.0 (2)
C6—C1—C10	121.1 (2)	C4—C5—H5	119.5
O2—C11—O1	123.5 (2)	C6—C5—H5	119.5
O2—C11—C12	125.1 (2)	C3—C2—C1	121.0 (2)
O1—C11—C12	111.4 (2)	C3—C2—H2	119.5
C8—C7—C6	110.88 (19)	C1—C2—H2	119.5
C8—C7—H7A	109.5	C2—C3—C4	119.5 (2)
C6—C7—H7A	109.5	C2—C3—H3	120.3
C8—C7—H7B	109.5	C4—C3—H3	120.3
C6—C7—H7B	109.5	C11—C12—H12A	109.5
H7A—C7—H7B	108.1	C11—C12—H12B	109.5
O1—C8—C7	112.88 (18)	H12A—C12—H12B	109.5
O1—C8—C9	106.93 (17)	C11—C12—H12C	109.5
C7—C8—C9	112.26 (19)	H12A—C12—H12C	109.5
O1—C8—H8	108.2	H12B—C12—H12C	109.5
			
C5—C6—C1—C2	1.2 (3)	C2—C1—C10—C9	−167.4 (2)
C7—C6—C1—C2	−178.7 (2)	C6—C1—C10—C9	13.3 (3)
C5—C6—C1—C10	−179.4 (2)	C2—C1—C10—Cl1	72.7 (2)
C7—C6—C1—C10	0.6 (3)	C6—C1—C10—Cl1	−106.6 (2)
C8—O1—C11—O2	3.4 (3)	C8—C9—C10—C1	−43.7 (3)
C8—O1—C11—C12	−175.2 (2)	Cl2—C9—C10—C1	77.0 (2)
C1—C6—C7—C8	17.2 (3)	C8—C9—C10—Cl1	78.3 (2)
C5—C6—C7—C8	−162.8 (2)	Cl2—C9—C10—Cl1	−161.00 (12)
C11—O1—C8—C7	−79.8 (2)	C3—C4—C5—C6	−0.2 (4)
C11—O1—C8—C9	156.3 (2)	C1—C6—C5—C4	−1.0 (3)
C6—C7—C8—O1	−170.16 (18)	C7—C6—C5—C4	179.0 (2)
C6—C7—C8—C9	−49.2 (3)	C6—C1—C2—C3	−0.3 (4)
C10—C9—C8—O1	−172.10 (18)	C10—C1—C2—C3	−179.6 (2)
Cl2—C9—C8—O1	68.8 (2)	C1—C2—C3—C4	−0.9 (4)
C10—C9—C8—C7	63.6 (2)	C5—C4—C3—C2	1.2 (4)
Cl2—C9—C8—C7	−55.5 (2)		

## References

[bb1] Blessing, R. H. (1995). *Acta Cryst.* A**51**, 33–38. 10.1107/s0108767394005726 7702794

[bb2] Farrugia, L. J. (1997). *J. Appl. Cryst.* **30**, 565.

[bb3] Farrugia, L. J. (1999). *J. Appl. Cryst.* **32**, 837–838.

[bb4] Frimer, A. A. (1985*a*). *Singlet Oxygen*, Vol. II, Part 1. Boca Raton: CRC Press.

[bb5] Frimer, A. A. (1985*b*). *Singlet Oxygen*, Vol. III, Part 2. Boca Raton: CRC Press.

[bb6] Kishali, N., Sahin, E. & Kara, Y. (2006*a*). *Org. Lett.* **2006**, 60, 1791–1793. 10.1021/ol060272s 16623552

[bb7] Kishali, N., Sahin, E. & Kara, Y. (2006*b*). *Helv. Chim. Acta*, **89**, 1246–1253.

[bb8] March, J. & Smith, M. B. (2001). *Advanced Organic Chemistry* New York: John Wiley and Sons.

[bb9] McBride, C. M., Chrisman, W., Harris, C. E. & Singaram, B. (1999). *Tetrahedron Lett.* **40**, 45–48.

[bb10] Metha, G. & Ramesh, S. S. (2003). *Tetrahedron Lett.* **44**, 3105–3108.

[bb11] Metha, G. & Ramesh, S. S. (2005). *Eur. J. Org. Chem.* pp. 2225–2238.

[bb12] Metha, G., Ramesh, S. S. & Bera, M. K. (2003). *Chem. Eur. J.* **9**, 2264–2272. 10.1002/chem.200204650 12772301

[bb13] Patai, S. (1983). Editor. *The Chemistry of Functional Groups, Peroxides.* New York: Wiley.

[bb14] Rigaku/MSC (2005). *CrystalClear* Rigaku/MSC, The Woodlands, Texas, USA.

[bb15] Ros, A., Magriz, A., Dietrich, H., Fernandez, R., Alvarez, E. & Lassaletta, J. M. (2006). *Org. Lett.* **8**, 127–130. 10.1021/ol052821k 16381584

[bb16] Sheldrick, G. M. (2008). *Acta Cryst.* A**64**, 112–122. 10.1107/S0108767307043930 18156677

[bb17] Wasserman, H. H. & Murray, W. M. (1979). Editors. *Singlet Oxygen* New York: Academic Press.

